# Suicidal Ideation Risk and Socio-Cultural Factors in China: A Longitudinal Study on Social Media from 2010 to 2018

**DOI:** 10.3390/ijerph18031098

**Published:** 2021-01-26

**Authors:** He Li, Yujin Han, Yunyu Xiao, Xingyun Liu, Ang Li, Tingshao Zhu

**Affiliations:** 1 Institute of Psychology, Chinese Academy of Sciences, Beijing 100101, China; lih@psych.ac.cn; 2 Department of Psychology, University of Chinese Academy of Sciences, Beijing 100049, China; 3 Department of Statistics, Renmin University of China, Beijing 100872, China; hanyujinruc@163.com; 4 School of Social Work, Indiana University–Purdue University Indianapolis, Indianapolis, IN 46202, USA; yx18@iu.edu; 5 School of Psychology, Central China Normal University, Wuhan 430079, China; liuxingyunpsy@163.com; 6 Department of Psychology, Beijing Forestry University, Beijing 100083, China; angli@bjfu.edu.cn

**Keywords:** suicidal ideation risk, socio-cultural, social media, word frequency, China, psychache, two-way fixed effect

## Abstract

Many studies cited the importance of social factors as protective and risk factors for suicide. However, there is a lack of evidence on the influences of cultural and moral values. This study aims to examine the association between cultural values and suicidal ideation risks detected on an online social media platform. We collected a total of 5.1 billion pieces of Weibo posts from 2010 to 2018 to calculate their suicidal ideation risks as measured by psychache in the Chinese Suicide Dictionary. We calculated the word frequencies of cultural and moral values based on Cultural Value Dictionary and Moral Foundation Dictionary. We collected economic and population data from the China National Bureau of Statistics. Two-way fixed-effect models were performed to analyze the association between culture, economy, and population factors and suicidal ideation risks. The results confirm the relations between high suicidal ideation risk and public concerns of vice under the Chinese context such as harm (*β* = 0.193, *p* < 0.01), betrayal (*β* = 0.096, *p* < 0.01), and dirty (*β* = 0.624, *p* < 0.001). In addition, extremely individualistic or collectivistic values of the public were associated with high suicidal ideation risks. The finding indicated the significant impact of social culture on suicide risk apart from the influence of the social economy and population characteristics. Our evidence informs population-based suicide prevention policymakers that incorporating cultural and moral values can help prevent suicidal ideation in China.

## 1. Introduction

Suicide is a serious public health problem in China and around the world. The number of suicide deaths was estimated to be more than 250,000 in China and approximately 800,000 in the world in 2014 [1,2]. From a social perspective, suicide mortality may increase the burden of disease and cause loss of productivity [3], calling for a public health reaction to prevent suicide. Identifying suicide risks is critical to improve the efficacy and effectiveness of preventative programs. Previous studies have suggested social factors related to suicide risk across populations [4]. However, such studies mainly focused on the mortality of suicide, but not nonfatal suicidal behaviors. Suicidal thoughts (defined as clear or unclear suicidal intent) are more common than suicide death and can predict future suicide and suicide attempts [5,6]. This study will focus on predictive factors related to suicidal thoughts.

Suicidality occurs on a continuum of progress from less serious to increasingly severe [7]. Relatively few people will die by suicide without disclosure of suicidal thoughts and planing [8], whereas many incidents are accompanied by severe negative mental states, such as psychache [9]. Shneidman (1993) introduced the concept “psychache” to describe an introspective experience with overwhelming feelings of “guilt, shame, humiliation or loneliness or fear or angst or dread of growing old or dying badly [10] (p. 145).” Previous studies revealed positive association between key domains of psychache (e.g., pain and vulnerability) and suicidal thoughts [11,12]. Relieving psychache may constitute a distinct and important treatment goal in the future [11]. The current study will use psychache as a proxy for suicidal thoughts.

Previous studies have discovered the link between social factors (e.g., macroeconomy [13] and population [14]) and suicidal behaviors, for example, finding a positive correlation between urbanization and suicide rate in American [15] and Finnish [16] contexts. However, studies are lacking in social culture influences, such as the regional differences in cultural values or moral values. It is widely accepted that social culture is a significant aspect of society that is capable of influencing mental health and behavior [17,18]. Cross-cultural studies suggested that subjects from different cultures displayed heterogeneity of suicide problems in terms of values, beliefs, attitudes, and other perspectives [19,20]. In addition, morality plays a unique role in restraining, regulating, and guiding the social life of the Chinese. Violating morality may increase self-prescribed stress as a result of feeling less satisfied in life and worrying about the negative consequences of betraying social norms [21,22]. The present study focuses on the impact of cultural and moral values on suicidal thoughts, in addition to social factors (see Table 1).

Previous studies typically used self-report or questionnaires to determine individuals’ suicidal thoughts and cultural tendencies [23,24]. However, such traditional methods generally limit sample size and require additional material and time investment, which may lead to less empirical research on social culture and suicidal thoughts that is longitudinal in nature with large samples. In recent years, many researchers used social media to measure social culture and suicide risk [25,26,27]. Social media can be another assessment tool for measuring several unobservable indicators within society.

In China, social media gradually became the main platform for sharing thoughts online since 2008, with an increasing number of users per year [28]. As of 2018, Weibo (a social media platform that is comparable to Twitter), one of the mainstream social media platforms in China, reached 462 million active users monthly [29]. The users mainly share text messages to express personal views in real-time and produce massive amounts of publicly available data at the same time. These data have been widely used for measuring suicidal ideation [26], moral values [25], and cultural values [30]. The study aims to explore the relationship between suicide risk and socio-cultural factors based on social media in China.

## 2. Materials and Methods

### 2.1. Data Collection

We used the Weibo public Application Programming Interface (API) to download the posts from 1 January 2010 to 31 December 2018 of all active users, which covered 31 provinces and autonomous regions in China. Active users were certified as non-institutional personal and non-overseas addresses. Those users had more than 500 posts after registering an account and had recently published Weibo posts [28]. All data used in research are publicly available, and the privacy of users was strictly protected, referring to the ethical principles [31]. The research methods and procedures adopted in this study were approved by the Research Ethics Committee of the Institute of Psychology, Chinese Academy of Sciences, ethic code is H15009.

For social and cultural factors, we collected variables identified as suicide risks in the previous literature, including population density [32], unemployment [33,34], geographic mobility [35], economic situation [36], divorce [37], and medical expenditure [38], as shown in Table 1. The indicators were obtained from the website of the China National Bureau of Statistics from 2010 to 2018 [39], the same time period as our social media posts.

### 2.2. Measurement of Suicidal Ideation Risk and Culture

#### 2.2.1. Dependent Variable: Suicidal Ideation Risks

The Chinese suicide dictionary compiled by Lv (2015) was employed [40]. Researchers obtained the corpus from Weibo posts with suicidal ideation risk and screened according to the strict exclusion criteria like ambiguity and low-frequency vocabulary [40]. These suicidal ideation risks were characterized by keywords of the sub-dimensions of “psychache.” The psychache dimension contains 403 Chinese keywords related to suicidal ideation risks on social media [40].

#### 2.2.2. Independent Variable: Social, Cultural, and Moral Factors

We used the Cultural Value Dictionary developed by Ren (2017) to calculate the collective tendency of individualism and collectivism on Weibo [30]. The dictionary was contributed and screened based on relevant vocabulary mentioned in previous cross-cultural studies [30]. Finally, it determined 53 individualistic and 64 collectivistic vocabularies in Chinese. That has been used to calculate the degree of collectivism in the early stage of the COVID-19 outbreak in China by the researcher [41].

The Moral Foundation Dictionary, revised by Wu (2019) to calculate the morality importance for the public [42]. The dictionary is translated and revised based on the Moral Foundation Dictionary established by Graham. The Chinese version dictionary contains 10 dimensions of moral foundation, and has been used by researchers to measure the moral tendency of Chinese citizens [25].

The details of the dictionaries are shown in Table 2.

We employed the “TextMind” system developed by the Computational CyberPsychology Laboratory at the Institute of Psychology, Chinese Academy of Sciences, to process the word segmentation of Weibo posts and calculate word frequency. The specific word frequency calculation process is the number of keyword appearances divided by the total number of posts words.

Then, we had achieved quantification of all indicators. In order to eliminate dimensional differences, it was necessary to standardize all the indicators by Z-score before entering the subsequent analysis.

Finally, we took the “year” and the “province (or autonomous region)” as the granularity and finally formed 9 (Time) and 31 (Cross-section), a total of 279 sets of panel data.

### 2.3. Statistical Analysis

This paper preferred to use a model with two-way fixed effects, which consider the potential information in both the cross-section and the time-series to which the observation belongs [43]. The model is given by:

Yit=βXit+λt+αi+εit

where Yit refer to Psychache estimated result at time *t*. Xit includes Care, harm, fairness, Fraud, Loyalty, Betrayal, Authority, Revolt, Purity, Dirty, Individualism, Collectivism, UPD, PC, CDR, R-GDP, URUR, LFMHE, LFTE, NBMI, and PTV. λt represents the time fixed effect, and represents the individual fixed effect. εit is the error term, which can vary over time and the fixed effect across the individual.

Although the two-way fixed effects model is theoretically the most suitable, it needs validations on data. First, we respectively constructed a construct model of mixed estimation, random effects, one-way fixed effects, and two-way fixed effects. Then, we used F-test and Hausman-test to select the best model successively. Finally, the test of cross-sectional correlation, autocorrelation, and heteroscedasticity was carried out in sequence to verify the reliability of the model and further optimized the model under the test result.

Further, we changed the measure of two independent variables to test the robustness of these estimates. The number of medical and health institutions (NMHI) was used instead of Number of Beds in Medical Institutions (NBMI) and registered urban unemployed population (RUUP) instead of Urban Registered Unemployment Rate (URUR). Since it is difficult to find another reliable method to calculate the social culture as replacement variables to re-estimate the model, the replaced variables all belonged to social-economic indicators and fit the granularity of the time and cross individual. All statistical analysis processes were using R public statistics software.

## 3. Results

### 3.1. Descriptive Statistic

**Figure 1:**
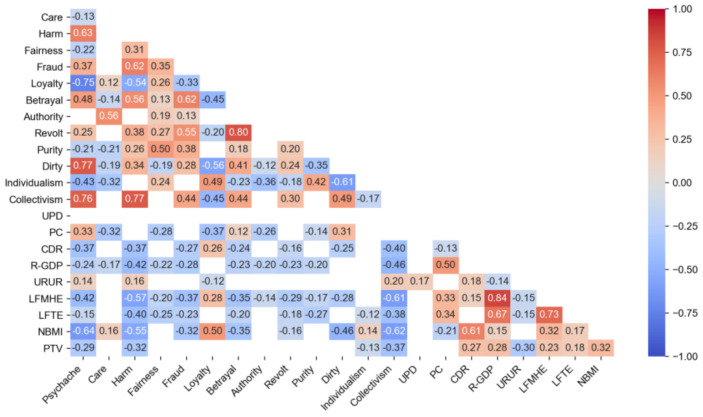
Variables correlation heat map. Null value means the correlation is not significant. Abbreviations: UPD, Urban population density; PC, Passenger capacity; CDR, Crude divorce rate; R-GDP, Regional gross domestic product; URUR, Urban registered unemployment rate; LFMHE, Local financial, medical and health expenditure; LFTE, Local fiscal transportation expenditure; NBMI, Number of beds in medical institutions; PTV, Public transportation vehicles.

According to the characteristic of all variables, they were divided into 3 dimensions. The descriptive statistic is shown in Table 3, and the correlation is shown in Figure 1.

### 3.2. Suicidal Ideation Risk on Social Media

**Figure 2:**
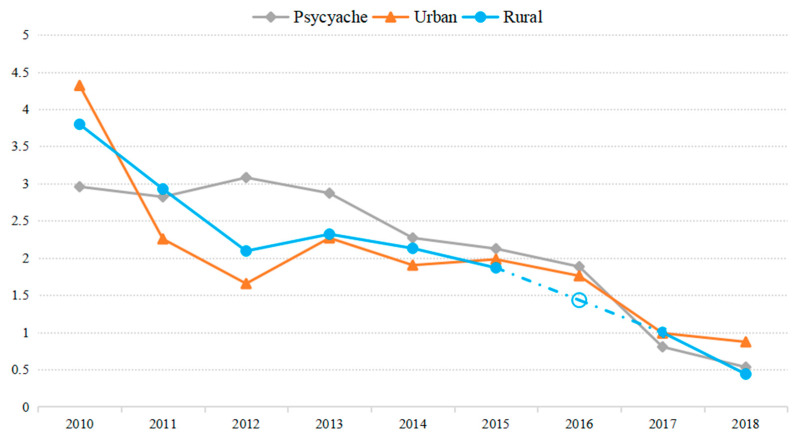
Trends of suicidal ideation risk on Weibo and social suicide rate, 2010–2018. Urban and Rural represent the suicide rate of urban or rural in China. Data are standardized by Z-score. The 2016 rural suicide rate was replaced by the before and after year (2015 and 2017) average value due to missing variables.

We analyzed the word frequency trends of psychache from 2010 to 2018 on Weibo (see Figure 2). In the past 9 years, the psychache score had been declining overall. It is roughly consistent with the trend of suicide mortality rate in China past decade and also confirms the feasibility of using social media to measure suicidal ideation risk.

Since 2010, China’s suicide rate has generally shown a slow downward trend, but there has been a slight rebound in 2013 [44]. The results of the study suggest the increase in suicidal ideation risk on Weibo in 2012 might be an early warning of this upward trend.

### 3.3. The Two-Way Fixed Effects Model

The F-test results of the psychache model are significant (*F* = 12.06, *p* < 0.001), which indicates that there exist unobservable individual differences inside. Secondly, the Hausman-test results shown that individual differences are related to independent variables (*Chisq* = 35.37, *p* = 0.026). Third, we evaluated the time-invariant characteristics of the fixed-effects model. Due to the Lagrange multiplier results are significant (*Chibarsq* = 153.96, *p* < 0.001), the two-way fixed-effects model is finally applied [45].

Further, we verified the reliability of the two-way fixed effects (FE) model by three statistical tests [45]. The cross-sectional correlation is not existing in the two-way FE model according to Pesaran’s CD Test (*Z* = −1.66, *p* = 0.098). Wooldridge’s test confirms there is serial autocorrelation inside (*F* = 10.77, *p* = 0.001). The Breusch-Pagan result shown that the two-way FE has heteroscedasticity (*BP* = 59.08, *p* < 0.001).

To solve the serial autocorrelation and heteroscedasticity problems within the two-way fixed effects model, we used the clustering robust standard error to correct the *t*-value [45], and the details are given in Table 4.

Further, we replaced two of the independent variables to verify the robustness of the model. The outcomes shown the coefficients and significance of the variables are basically unanimous in two models, which confirms the robustness of the model. The model outcomes of replacing the variables are shown in Table 5.

## 4. Discussion

Based on the variables covered in China’s context, the study found that social culture had a greater impact on suicidal ideation risk compared with the macroeconomy and population indicators. The influence of culture on people not only appears in “observable artifacts” but also in “underlying assumptions.” Although such influences are unconscious, they determine how people think, feel, and act [46].

The study revealed two social-culture factors related to suicidal ideation risks in China. First, a positive correlation was observed between suicidal ideation risk and vice moral dimension (i.e., harm, betrayal, and dirty). Chinese society advocates positive morality, whereas negative morality violates mainstream values [47]. The study finds that when large-scale social anomie posts exist, the risk of social suicide increases [48]. Second, cultural values with strong individualistic or collectivistic characteristics are related to high suicidal ideation risk. This finding is in agreement with those verified by previous studies from the public perspective [17]. Different cultural values pose no advantages or disadvantages. For instance, collectivism may enhance social cohesion and promote social support, whereas individualism may enable people to express themselves better. Conversely, extreme individualism or collectivism may be prejudicious to the possible benefits of the two cultural values [17]. Specific discussions of the impact of social-culture on suicidal ideation risk are presented as follows.

Harm. A high degree of harm emphasis may imply a high risk of violence. Many similarities are observed between suicide and violence [49], which are likely derived from the same potential aggression [50]. The emphasis on harm may be related to an unstable and violent social environment, such as frequent occurrences of crimes and major disasters, and which can also influence suicidal ideation risk [51,52,53].

Loyalty and betrayal. Both are a set of opposite public moral emphases, in which loyalty reflects a loyalty to a family or group and the virtues of patriotism [54]. Betrayal emphasizes outgroups and is a manifestation of abandonment of trust and responsibility. Numerous expressions of loyalty posted on social media may indicate that society is cohesive. On the contrary, when expressions of betrayal increase, the social connection may be negatively affected, which indicates a high risk of suicide. This finding is consistent with that of Durkheim (1951), indicating the degrees between political and social integration and between family and social integration are inversely proportional to suicide [55]. This notion may imply that building a cohesive society with the concept of loyalty has a positive effect on suicide.

Authority and revolt. Both terms describe the relationship between subordinates and authorities (e.g., obedience versus rebellion), which reflects the human hierarchy’s attention [54]. Since China’s unique civilization, the hierarchy was a respected construct and distinct within families (e.g., young versus old) and social class (e.g., leaders versus subordinates). This aspect established compact but distinct social relations. Previous studies proposed that hierarchy is harmful [56] or ineffective [57] in maintaining social health. However, the results from this stream of research indicate that group hierarchy may be a protective factor for suicide.

Dirty. The term refers to not only material pollution but also human spiritual desires, such as greed and selfishness [54]. The words used to pertain to dirty are derogatory. Whether they are used to describe an object or person, woods in within this context with strong derogatory implications, this phenomenon will not only destroy peace and harmony in society but also harm individuals in the society. The present research implies that when dirty messages frequently occur, significantly high suicidal ideation risk was observed.

Individualism and collectivism. The tendency of individualism indicates more distant social connections and less social support networks, which may render dealing with negative life events difficult for many individuals [58,59]. Conversely, cross-cultural research implies that the value orientation of young adults is in contrast to the social environment. Seemingly, young adults in individualist countries intend to communicate and connect with society, whereas peers in collectivist countries aim to maintain individuality [17]. China is recognized as a traditional collectivist country [60]. However, young adults pay more attention to personal development and feeling, which benefited from the high quality of life and educational resources brought about by the rapid development of the economy [61]. However, the previous generation remains in power, whether in society, among groups, or within the family [62]. Thus, the conflict in cultural values between the two generations may be one of the causes of suicidal thoughts.

In terms of social conditions, public transportation leads to a positive effect on suicidal ideation risk. The number of public transportation vehicles is an important indicator for measuring the progress of new urbanization [63]. Many studies based on the context of western countries point out that urbanization level and suicide rate increase in a positive proportion [15,16]. However, this rule does not appear suitable for the Chinese context.

Public transportation vehicles. As a major infrastructure, public transportation vehicles are largely an urban population carrier, which has grown with the process of urbanization. In addition, Ye (2014) argued that urbanization reduces the suicide rate because it promotes population migration, where the urban middle class is increasing [64]. The current study verified the results of previous studies based on the Chinese context.

The present study explored social factors related to suicidal ideation risk on Weibo. We found that social culture has an important influence on suicidal ideation risk. Person who took their own life does not only exist individual reasons but also due to social reality. In China, the unique contribution of moral and cultural values to suicidal ideation risk is undeniable because the feudal rule history in China and development in modernization are unique, which has a large impact on the cognition and behavior of the Chinese. Our findings can inform policymakers in designing structural interventions that target moral and cultural values in society to prevent the occurrence of suicidal thoughts. Suicide is a global problem; thus, suicidal ideation risk factors in Chinese society may also exist and influence other countries. The results could provide references to other countries and regions.

The study has limitations. First, the research subjects were largely derived from social media. The current status of the Chinese Internet age structure is that users aged 10 to 39 years account for 61.6% of all Internet users [65]. Although the problem of suicide is more serious among young adults in China, social media users cannot represent all Chinese people due to the lack of data on the elderly. Second, the results may be more applicable to the virtual Internet society because information on social culture and suicidal ideation was extracted from social media. However, online and offline situations may differ. Third, our word frequency analysis among the extracted variables accounted for the word counts but not related contextual factors behind each word. This limits the explanatory power of the variables. Future studies should review the vocabulary words in the context of individual cases [66]. Finally, although psychache is associated with the risk of suicidal ideation, future research need to conducted to identify relationship between psychache and suicide thoughts of real Weibo users. This aspect will be an interesting avenue for future research.

## 5. Conclusions

The present study focused on socio-cultural factors that influence suicide risk. The word frequency ratio of suicide risk and cultural and moral values information derived from Weibo was calculated. We constructed a two-way fixed effect model to achieve the purpose of the experiment. The outcomes confirmed that socio-cultural is closely related to suicide. An unethical environment may lead to more suicide risks. Furthermore, overly individualistic or collectivistic values were deemed detrimental to mental health. This evidence could provide a certain degree of enlightenment for suicide prevention from the public perspective. In addition to individual prevention, macro-control from the sociocultural perspective can also be used to reduce the suicide risk by osmosis. Although comprehending suicide from the cultural perspective is meaningful, the study lacked depth, which requires further complex analysis methods for exploring the specific impact pattern between social culture and suicide.

## Figures and Tables

**Table 1:** Definitions of social economy and the population indicators, 2010–2018.

Variables	Indicators	Definitions
UPD	Urban Population Density (person/sq.km)	The sum of the urban population and the temporary population is divided by the urban area.
PC	Passenger Capacity (per 10,000 people)	Refers to the number of passengers actually transported by various means of transportation within a certain period of time.
CDR	Crude Divorce Rate (‰)	The ratio of the annual number of divorces in the region to the average total population.
R-GDP	Regional Gross Domestic Product (100 million yuan)	The sum of the production activities of all resident units in a region within a certain period of time.
URUR	Urban Registered Unemployment Rate (%)	The proportion of the number of registered urban unemployed persons and the sum of the number of urban employed and unemployed persons.
LFMHE	Local Financial Medical and Health Expenditure (100 million yuan)	Refers to government medical and health expenditure, including medical service expenditure, medical security expenditure, disease prevention, and control expenditure, etc.
LFTE	Local Financial Transportation Expenditure (100 million yuan)	Reflect the local government expenditure on transportation, including road, railway, and civil air, etc.
NBMI	Number of Beds in Medical Institutions (sheets/per 10,000 people)	The number of beds in medical and health institutions divided by population and multiplied by 10,000.
PTV	Public Transportation Vehicles (units/per 10,000 people)	The number of public transportation vehicles divided by the sum of urban population and temporary residents in urban areas.

**Table 2:** Detailed description of the dictionaries.

	Category	Number of Words	Definitions	Representative Words
Moral Foundation Dictionary	Care	38	Protect and help others	Care (关爱) Protection (保护)
	Harm	79	Injuries and violent behavior	Detriment (危害) Damage (损伤)
	Fairness	36	Equal social relations	Fairness (公平) Average (平均)
	Fraud	32	Cheating and unfair phenomenon	Fraud (欺骗) Favoritism (偏袒)
	Loyalty	70	Ingroup and cooperation	Team (团体) Alignment (结盟)
	Betrayal	51	Outgroup and betrayal	Betrayal (背叛) Illegal (违法)
	Authority	81	Hierarchical structure within the organization	Compliance (服从) Responsibility (责任)
	Revolt	46	Rebel against authority	Provocation (挑衅) Defy (违抗)
	Purity	58	Pure body and mind	Sterility (无菌) God (上帝)
	Dirty	99	Dirty body and mind	Pollution (污染) Evil (邪恶)
Cultural Values Dictionary	Individualism	53	Represent self-attentional focus	Independence (独立) Individuality (个性)
	Collectivism	64	Represent attentional focus toward others or groups	Coordination (协调) Interaction (互动)
Chinese Suicide Dictionary	Psychache	403	Psychological distress	Miserable (凄苦) Grief (悲恸)

**Table 3:** Descriptive statistics for variables, 2010–2018.

	Variables	Mean	SD
Suicidal ideation risk	Psychache	4.88	1.10
Social Culture (10^−2^)	Care	0.11	0.02
	Harm	0.48	0.08
	Fairness	0.13	0.02
	Fraud	0.07	0.02
	Loyalty	1.81	0.42
	Betrayal	0.13	0.04
	Authority	0.62	0.13
	Revolt	0.11	0.03
	Purity	0.39	0.05
	Dirty	0.47	0.20
	Individualism	22.06	3.82
	Collectivism	7.99	0.71
Population	Urban population density	2797.77	1178.13
	Passenger capacity	79,020.77	74,419.36
	Crude divorce rate	2.70	1.00
Economy	Regional gross domestic product	21,929.80	18,146.08
	Urban registered unemployment rate	3.31	0.65
	Local financial, medical, and health expenditure	327.03	220.66
	The local fiscal transportation expenditure	277.67	178.21
	Number of beds in medical institutions	48.62	10.63
	Public transportation vehicles	12.36	3.27

**Table 4:** Regression outcomes of two-way FE model of clustering robust standard errors.

Variables	Psychache	
	Estimate (10^−2^)	Clustering Robust SE (10^−2^)
UPD	−3.22	4.52
PC	1.31	5.60
CDR	−4.52	3.87
R-GDP	−12.86	8.35
URUR	0.69	3.49
LFMHE	−8.23	8.42
LFTE	1.65	2.35
NBMI	5.10	3.03
PTV	7.98 *	3.52
Care	−1.05	4.39
Harm	19.31 **	6.15
Fairness	4.52	3.16
Fraud	2.20	2.14
Loyalty	−26.04 **	8.13
Betrayal	9.62 **	3.51
Authority	−6.52 *	3.28
Revolt	−6.90 *	3.09
Purity	2.38	3.51
Dirty	62.43 ***	7.96
Individualism	38.50 ***	9.18
Collectivism	26.16 ***	6.23
R^2^	0.62	

Abbreviations: UPD, Urban population density; PC, Passenger capacity; CDR, Crude divorce rate; R-GDP, Regional gross domestic product; URUR, Urban registered unemployment rate; LFMHE, Local financial, medical and health expenditure; LFTE, Local fiscal transportation expenditure; NBMI, Number of beds in medical institutions; PTV, Public transportation vehicles. *** *p* < 0.001, ** *p* < 0.01, * *p* < 0.05.

**Table 5:** Regression outcomes of two-way FE model of clustering robust standard errors with replacement variables.

Variables	Psychache	
	Estimate (10^−2^)	Clustering Robust SE (10^−2^)
NMHI	36.17	26.37
RUUP	−4.57	5.96
UPD	−4.60	4.51
PC	2.76	5.81
CDR	−1.01	4.18
R-GDP	−18.33	9.81
LFMHE	−4.61	8.27
LFTE	1.63	2.46
PTV	7.10 *	3.42
Care	−0.99	4.51
Harm	18.46 **	6.19
Fairness	5.08	3.17
Fraud	2.44	2.14
Loyalty	−28.62 ***	7.96
Betrayal	9.80 **	3.53
Authority	−7.19 *	3.22
Revolt	−30.73 *	3.09
Purity	2.15	3.47
Dirty	61.27 ***	8.17
Individualism	37.41 ***	8.92
Collectivism	24.85 ***	6.19
R^2^	0.51	

Abbreviations: UPD, Urban population density; PC, Passenger capacity; CDR, Crude divorce rate; R-GDP, Regional gross domestic product; URUR, Urban registered unemployment rate; LFMHE, Local financial, medical and health expenditure; LFTE, Local fiscal transportation expenditure; NBMI, Number of beds in medical institutions; PTV, Public transportation vehicles. *** *p* < 0.001, ** *p* < 0.01, * *p* < 0.05.

## Data Availability

Restrictions apply to the availability of these data. Data was obtained from Weibo and are available at (https://weibo.com) with the permission of Weibo.
